# Supplementary material for: Low-Pass Genome Sequencing-Based Detection of Paternity: Validation in Clinical Cytogenetics
Source: Genes (Basel). 2023 Jun 27;14(7):1357. doi: 10.3390/genes14071357 (PMC10379141; doi:10.3390/genes14071357)
Supplement: Supplementary file 1 [file genes-14-01357-s001.zip › Supplementary Materials.pdf]

Supplementary Materials

Supplementary Methods

Algorithm for paternity/maternity testing

The principle of paternity/maternity testing is to affirm the paternity/maternity inclusion or exclusion according to the range of calculated paternity index. In the algorithm, an "inconsistent rate of base-type inheritance" between the proband and the presumed parent is used as paternity (or maternity) index for paternity (or maternity) confirmation.

Two analytical models are presented in our pipeline: a duo mode and a trio mode. For the trio-based analysis mode, loci in which both parents were homozygous for different genotypes were selected (for instance, a locus where the father was with homozygous A, whereas the mother was with homozygous T). In theory, the proband should carry a heterozygous AT genotype. However, in low-pass GS setting, proband could also show a homozygous genotype similar to one of parents (**Supplementary Figure S1A**). It might be due to: (a) one of the parents had a heterozygous genotype but mistakenly assigned as homozygous; (b) proband was detected as heterozygous but mistakenly assigned as homozygous; or (c) the genotype in one of the parents was resulted from systematic error(s). Lastly, the proband may carry a heterozygous genotype (*e.g.,* AG) but one base type (*i.e.,* G) was from neither parent. In addition to these false SNV calling events, the main reason for the inconsistency of base-type inheritance between the proband and the presumed parent(s) was non-paternity and/or non-maternity. Therefore, we hypothesized that for paternity test (or maternity test), the inconsistent rate of base-type inheritance in a non-paternity (non-maternity) would be significantly higher than that in a biological family. The algorithm was

designed as follows (using paternity test as example): (I) the number of loci that both parents were in homozygous manner but with different genotypes in the i-th chromosome was denoted as A^i; (II) among them, the number of SNVs that were homozygous in the proband but with different genotypes from the presumed father was denoted as pi (mi: for maternity test); (III) the inconsistent rate of paternal inheritance αi in chromosome i was calculated as the formulas (1).

$$\alpha i = \frac{pi}{Ai} \quad (1)$$

(IV) the paternity was determined by using the average rate $\bar{\lambda}pat$ across all autosomal chromosomes as the formulas (2).

$$\bar{\lambda}pat = \frac{\sum_{i=1}^{n=22} \alpha i}{22} \quad (2)$$

The same as for maternity test, the inconsistent rate of maternal inheritance βi in chromosome i was calculated as the formulas (3), while the maternity was determined by using the average rate $\bar{\lambda}mat$ across all autosomal chromosomes as the formulas (4).

$$\beta i = \frac{mi}{Ai} \quad (3)$$

$$\bar{\lambda}mat = \frac{\sum_{i=1}^{n=22} \beta i}{22} \quad (4)$$

For the duo-based analytical mode, we hypothesized that in a locus, if it was homozygous in the presumed father/mother, in the proband, it was heterozygous with one allele identical with that of the parent or homozygous that was the same as the submitted parent. However, in low-pass GS setting, it might be homozygous in the proband, but the genotype was different from the parent potentially due to: (a) it was heterozygous in that parent but mistakenly assigned as homozygous;

or (b) it was heterozygous in the proband but mistakenly assigned as homozygous; or (c) the genotype in one of them was resulted from systematic error(s). In addition to these false SNV calling events, the main reason for the inconsistent base-type inheritance between the proband and the presumed parent was non-paternity and/or non-maternity. Therefore, we only selected those loci that both samples were in homozygous manner (green frames in **Supplementary Figure S1B**) and calculated the rate that they were with different genotypes (blue frames in **Supplementary Figure S1B**). The algorithm was designed as follows (using paternity test as example): (I) the number of homozygous SNVs in both proband and the presumed parent in chromosome i is denoted Adi; (II) among them, the number of homozygous SNVs that were with different genotypes between the proband and the presumed father was denoted as qi; (III) the inconsistent rate γi of paternal inheritance in chromosome i was calculated as the formulas (5).

$$\gamma i = \frac{qi}{Adi} \tag{5}$$

(d) the paternity was determined by using the average rate $\bar{\lambda} pat$ across all autosomal chromosomes as the formulas (6).

$$\bar{\lambda} pat = \frac{\sum_{i=1}^{n=22} \gamma i}{22} \tag{6}$$

The maternity was determined with the same method as paternity determination.

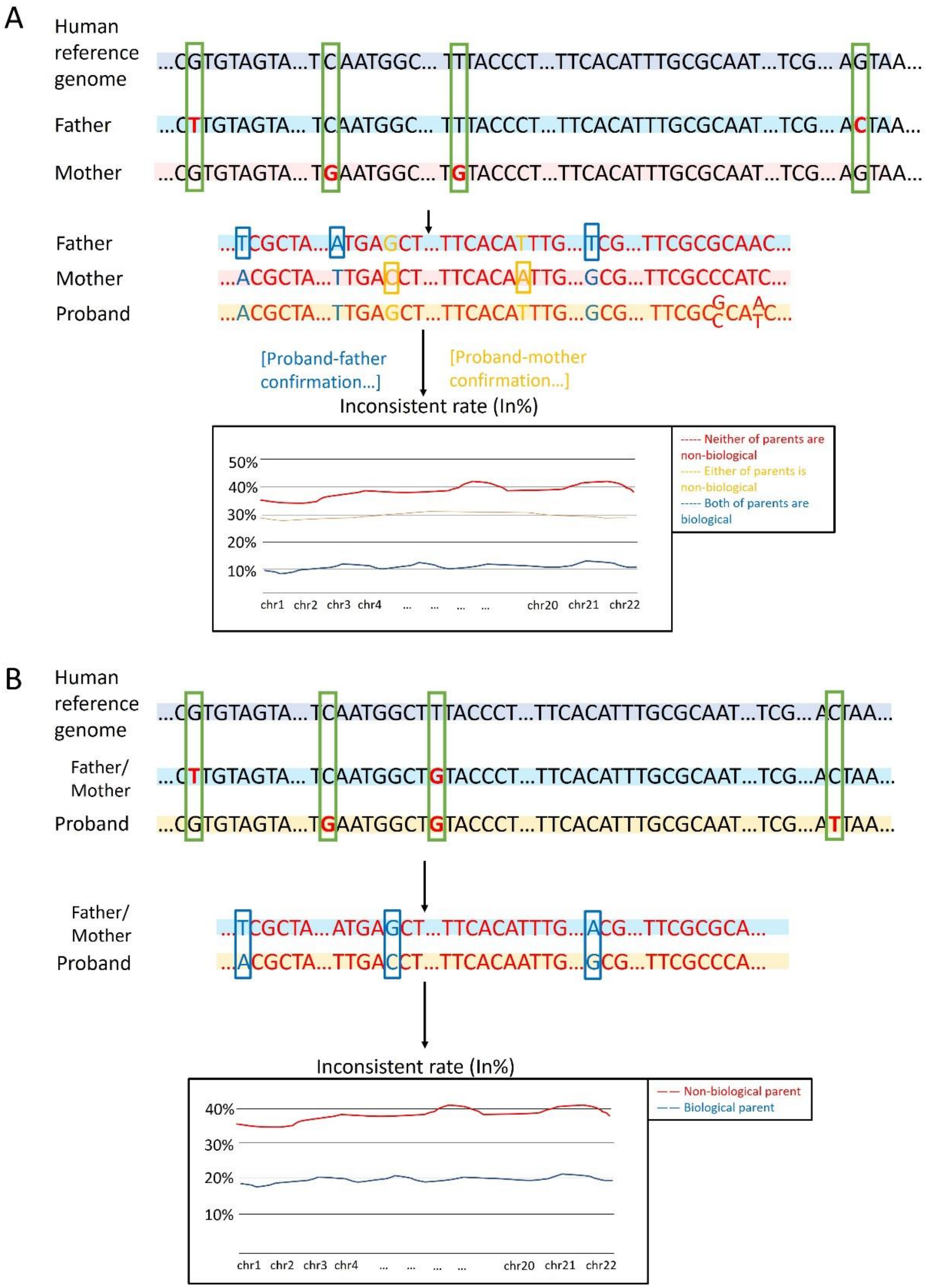

Supplementary Figure S1. Diagram of paternity testing by low-pass GS in two analytical modes. (A) Paternity/maternity testing in trio-based analysis. In the upper panel, loci that are homozygous in both parents but with different genotypes are indicated by green frames. In the

middle panel, those loci that with inconsistency of paternal or maternal inheritance are shown in blue or orange frames. The inconsistent rate of base-type inheritance is shown in the bottom. (B) Paternity/maternity testing in trio-based analysis. In the upper panel, loci that are homozygous in the proband and the presumed father/mother are indicated by green frames. In the middle panel, those loci that with inconsistency of paternal/maternal inheritance are shown in blue frames. The inconsistent rate of base-type inheritance is shown in the bottom.

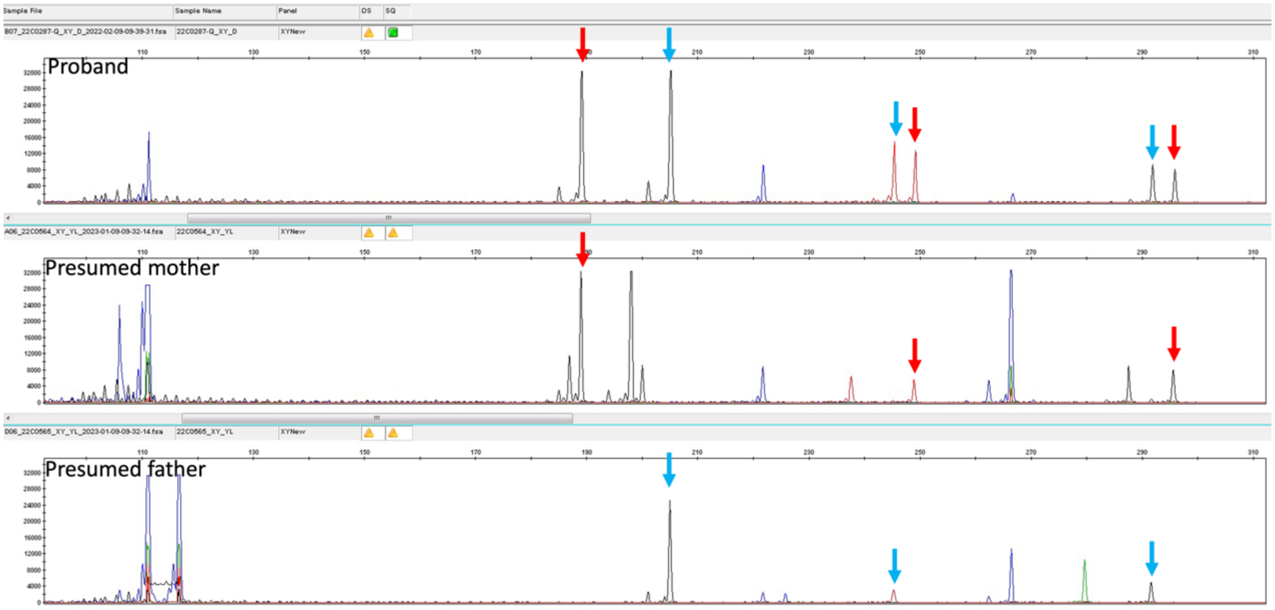

Supplementary Figure S2. Biological relationship among the trios confirmed by QF-PCR. QF-PCR with STR marker in family of 22C1246 (proband), 22C1607 (the presumed mother), and 22C1608 (the presumed father) is shown in the upper, middle and bottom panel, respectively. Each pair of a locus is linked, the allele in the proband inherited from the father is indicated by a blue arrow, while the other allele in the proband inherited from the mother is indicated by a red arrow.

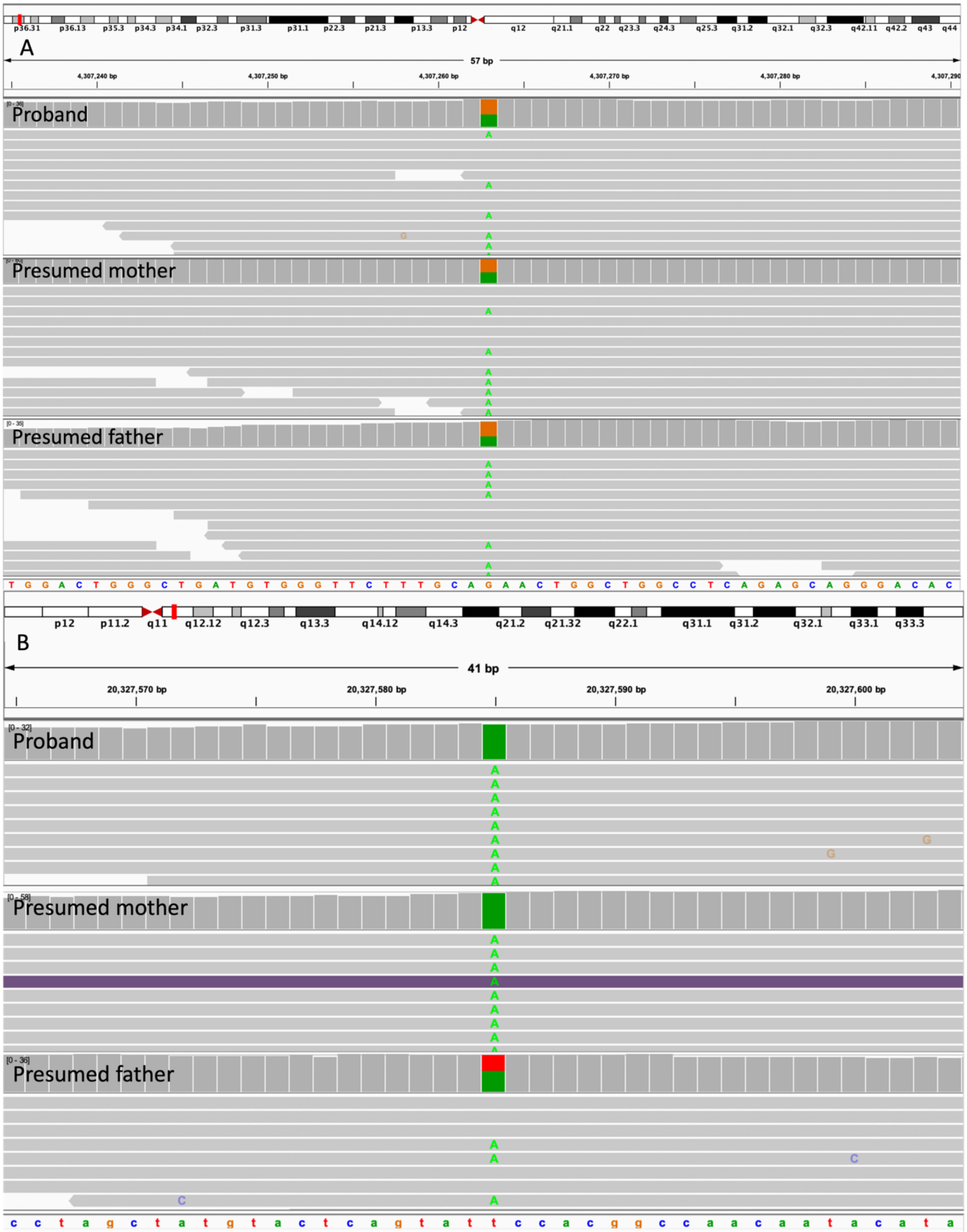

Supplementary Figure S3. Confirmation of biological relationship by comparing the genotypes among the proband and the presumed parents from the 1000 Genomes Project.

Integrative Genomics Viewer view of genotypes among the proband (upper panel), the presumed mother (middle panel) and the presumed father (lower panel). (A) the locus is rs1490413 with location of 4307263 in hg38 and of 4367323 in hg19. (B) the locus is rs1335873 with location of 20327585 in hg38 and of 20901724 in hg19.

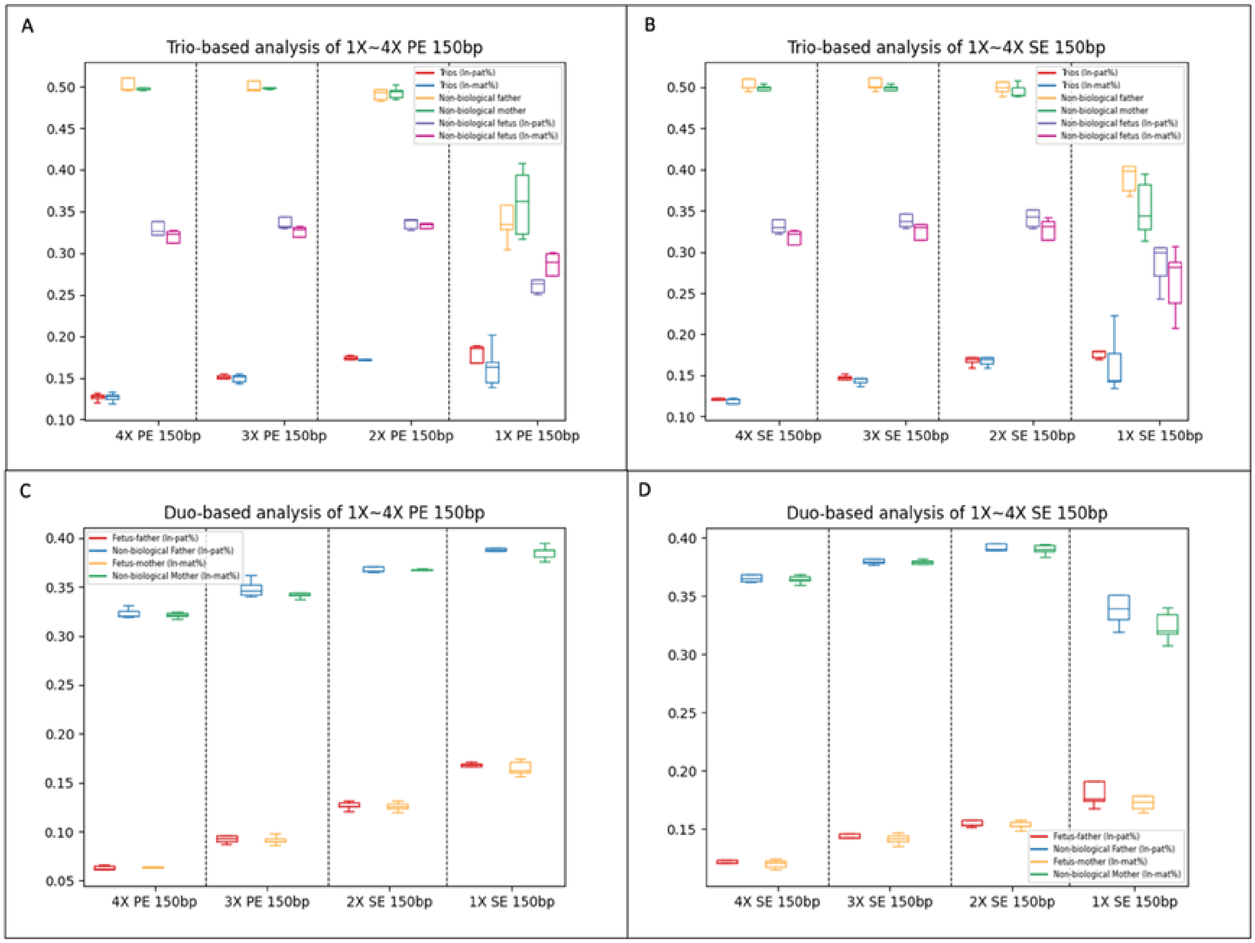

Supplementary Figure S4. Determination of the optimal read-depth required for the analysis. Boxplot of the inconsistent rate of parental inheritance among data with different read-depths with small insert libraries and paired-end 150bp in trio-based analysis (A) and in duo-based analysis (B), or single-end 150bp in trio-based analysis (C) and in duo-based analysis (D).

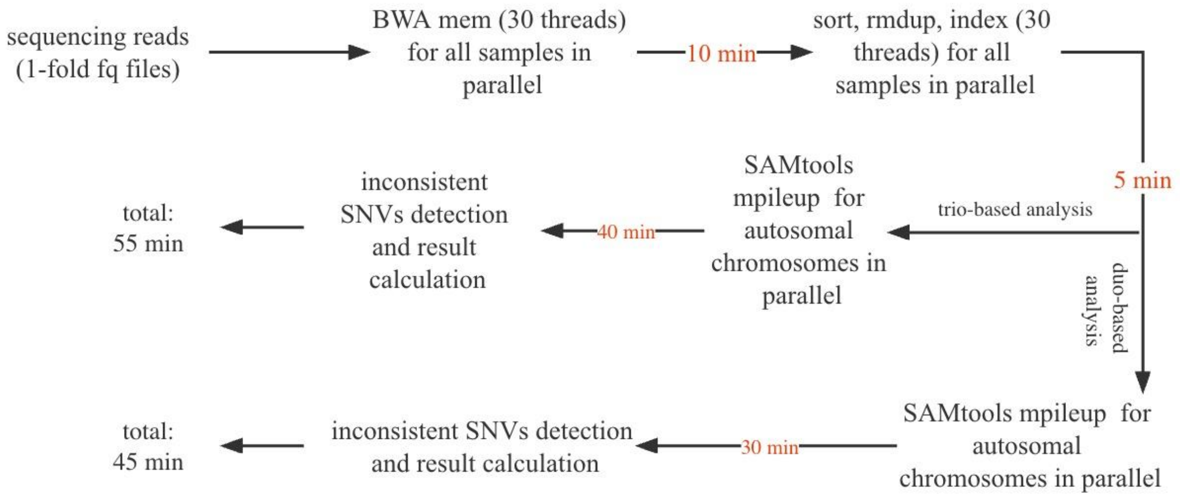

Supplementary Figure S5. The analytical process and turn-around-time estimation. The upper panel indicates the shared procedures between two modes, the rest of procedures in trio-based and duo-based analysis are shown in the middle or bottom, respectively. Each font in red indicates the average turn-around-time for that procedure.

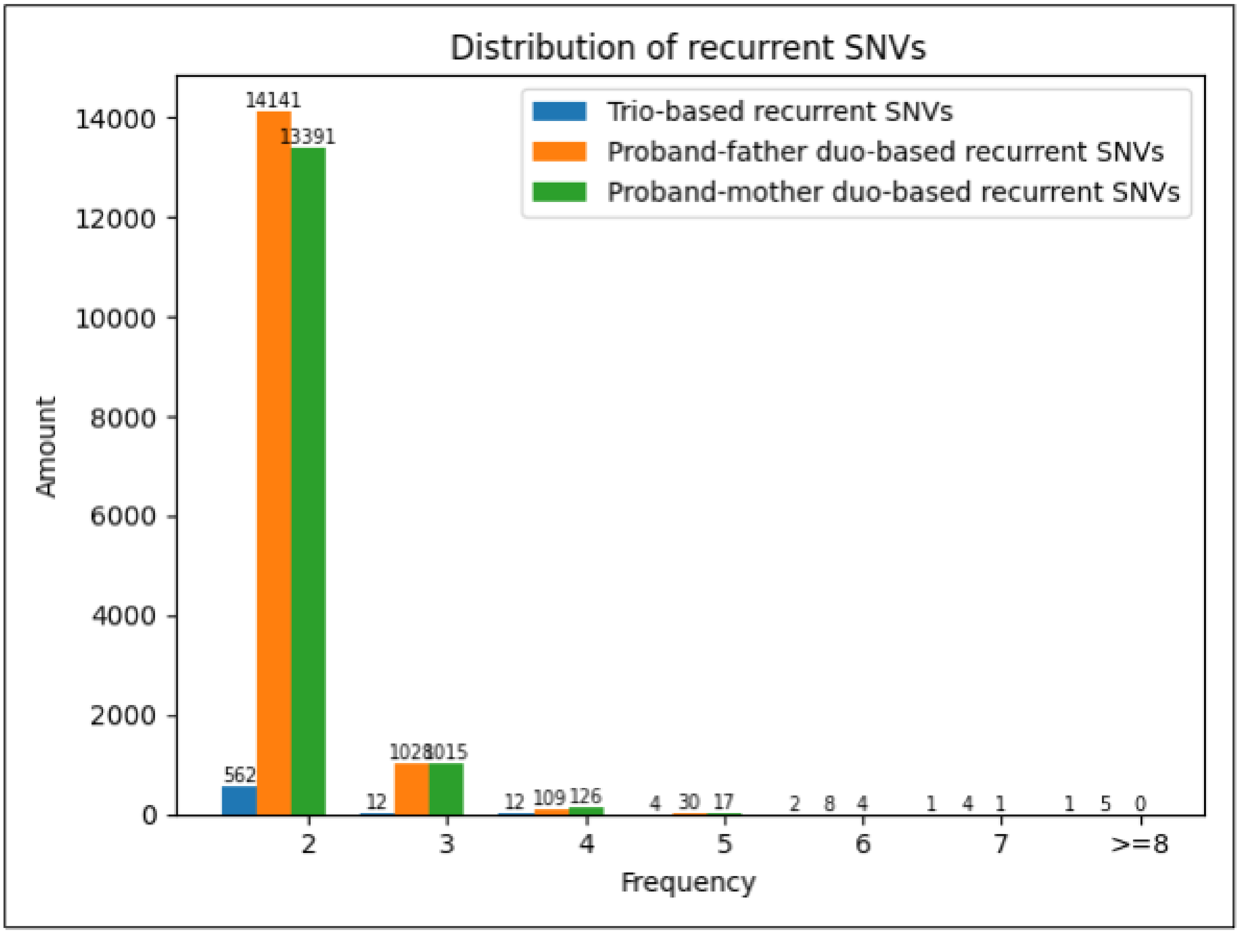

Supplementary Figure S6. Frequency distribution of the recurrent SNVs detected in trio-based and duo-based analysis. X axis indicates the number of detected and the Y axis shows the number of recurrent SNVs.
